# Effect of compassion fatigue on emotional labor in female nurses: Moderating effect of self-compassion

**DOI:** 10.1371/journal.pone.0301101

**Published:** 2024-03-28

**Authors:** Li-Chuan Chu

**Affiliations:** 1 School of Health Policy and Management, Chung Shan Medical University, Taiwan, Republic of China; 2 Department of Medical Education, Chung Shan Medical University Hospital, Taiwan, Republic of China; Zhejiang Shuren University, CHINA

## Abstract

Emotional labor is common in nursing but may be affected by the mental state of nurses. This study explored the effect of compassion fatigue on emotional labor and whether self-compassion moderates this effect of compassion fatigue. Methods: A two-stage survey design with a convenience sample. Participants were female nursing staff recruited from emergency departments, intensive care units, ward nursing units, and outpatient departments of medical centers, regional hospitals, and district hospitals in Taiwan. A total of 300 questionnaire copies in each of the first and second stages were distributed, and 272 pairs of responses were retrieved (valid response rate = 91%). The reliability and validity of the questionnaire were tested, and confirmatory factor analysis was conducted with AMOS 21. The proposed hypotheses were verified using hierarchical regression conducted with SPSS version 25.0. Results: This study revealed that compassion fatigue positively predicted surface acting (*β* = 0.12, p < 0.05) and negatively predicted deep acting (*β* = −0.18, p < 0.01) and expression of genuine emotions (*β* = −0.31, p < 0.01). In addition, self-compassion negatively moderates the relationships between compassion fatigue and surface acting (*β* = −0.12, *p* < 0.05), and positively moderates the relationships between compassion fatigue and expression of genuine emotions (*β* = 0.15, *p* < 0.01). Conclusions: To avoid excessive consumption of emotional resources, nurses with high compassion fatigue may employ surface acting by engaging in emotional labor without making an effort to adjust their feelings. Nurses need also be sympathized with, and such sympathy can come from hospitals, supervisors, colleagues, and, most crucially, the nurses themselves. Hospital executives should propose improvement strategies that can prevent the compassion fatigue on nurses, such as improving nurses’ self-compassion.

## Introduction

In high-stress nursing environments, nurses face challenges such as high workload, long working hours, lack of sleep, shift work, and dealing with various patient symptoms and emergencies [1,2]. These persistent stress factors can easily lead to occupational fatigue among nurses [3], often causing them to feel weak and burnt out. This fatigue can also impair their concentration on work tasks, negatively affect their job performance, increase the likelihood of care errors, and potentially endanger patient safety [2,4]. Occupational fatigue encompasses multiple dimensions, including the physical (lack of energy), cognitive (attention deficits), and emotional (decreased motivation or interest) dimensions [4].

Compassion is often seen as a crucial trait in nurses [5], yet the stress from prolonged compassionate engagement can easily lead to compassion fatigue [6]. Compassion fatigue, due to long-term exposure to patients’ suffering and excessive emotional involvement, causes nurses to appear indifferent and numb [7]. As a result, nurses experience difficulties managing and expressing appropriate emotions when interacting with patients or their families. In other words, compassion fatigue may influence nurses’ emotional labor, which is defined as the demand for expressing the appropriate emotions in workplace situations [8].

However, most researchers studying the relationship between compassion fatigue and emotional labor have considered compassion fatigue as the cost of emotional labor, believing that emotional labor increases compassion fatigue and affects the commitment of nurses to display compassion and care for patients, which is ultimately detrimental to patient care [9,10]. Can compassion fatigue also affect the emotional labor of nurses? When nurses experience compassion fatigue, does the lack of internal resources cause nurses to adopt surface acting rather than deep acting or expression of genuine emotions when performing emotional labor?

Because few studies have focused on the effect of compassion fatigue on emotional labor, this study explored the topic from the perspective of conservation of resources theory and self-determination theory. In addition, self-compassion, defined as the support that one gives themself when experiencing pain [11], is regarded as an internal personal resource [12], and whether self-compassion moderates the influence of compassion fatigue on emotional labor was explored.

### Relationship between compassion fatigue and emotional labor

Hochschild [8] defined emotional labor as personal emotional management that enables individuals to create a suitable facial expression or physical movement observable to the public. When interacting with customers during work, employees tend to display the emotions expected by their organization to ensure high customer satisfaction. Surface acting and deep acting are the most common forms of emotional labor [8]. When employees use surface acting, their external emotions differ from their internal perceptions; they adjust only their external expression without changing their internal feeling. When employees perceive a difference between their feelings and those expected by the organization, emotional dissonance occurs. By contrast, employees who use deep acting attempt to perceive the emotions they are required to express. By adjusting their internal feelings, they ensure that their internal and external feelings are consistent. This reduces emotional dissonance [13]. A third form of emotional labor was identified by Ashforth and Humphrey [14], namely the expression of genuine emotions. This form of emotional labor is often observed in service sector workers, who naturally and voluntarily display the emotions expected by the public and their organization. Such employees do not need to deliberately exhibit a particular emotion; they can spontaneously and naturally express the emotions required by their organization.

Previous studies have discovered that surface acting can increase stress, burnout, negative emotions, and turnover intention of nurses [15–17], and reduce the work engagement [18]. By contrast, those who engaged in more deep acting had more positive emotional experiences, job satisfaction and improve service quality [16,18]. When nurses let out their true emotions spontaneously, their burnout was effectively reduced [15].

In contrast to Wang et al. [10], who determined that compassion fatigue is the cost of emotional labor, the present study delved into the effect of compassion fatigue on emotional labor. Compared with emotional labor performed by general workers in the service sector, health care service providers generally choose their profession because of their sympathetic nature, altruistic motivation, or identification with the ideal that nurses should be sympathetic and compassionate toward patients. Therefore, nurses strive to help patients and genuinely express a sympathetic attitude toward patients [19]. Ideally, the emotional labor performed by nurses is mostly deep acting or the expression of genuine expression [19]. However, the emotional labor of nurses may be affected by various external limitations, or nurses may have limited ability to express a sympathetic attitude (e.g., compassion fatigue that is commonly observed in nurses). Whether nurses perform surface acting rather than deep acting as a result of compassion fatigue requires clarification.

Compassion fatigue is the stress accumulated within nurses over time due to their provision of prolonged care, support, and assistance to others, leading to emotional, physiological, and psychological exhaustion [20,21]. The concept of compassion fatigue was first proposed by Joinson [22]. When nurses in emergency departments or intensive care units face a sudden event such as a patient’s disease deterioration or death, they perceive themselves as unable to help and undergo complex emotional changes that lead to symptoms such as frequent headache or stomachache, reduced concentration, fatigue, depression, low pressure tolerance, irritability, indifference, and low work efficiency. The term compassion fatigue was formally introduced by Figley [20] to describe these symptoms. The emergence of compassion fatigue is attributed to nurses focusing excessively on patients’ suffering and the associated feelings of guilt and sorrow when they are unable to save the patients from their pain. The accumulating pressure causes them to question their ability when encountering patients with similar conditions. Because of a lack of confidence, these workers gradually develop an indifferent attitude, which helps them escape from reality. In turn, this reduces their compassion toward patients and their passion for their work. Consequently, compassion fatigue is considered a cost of sympathetic attitude [20]. Coetzee and Klopper [23] argued that compassion fatigue was a developing process, and its development results from caregivers’ long-term exposure to stress caused by their continuous and intensive contact with patients. The process starts from compassion discomfort, proceeds to compassion stress if appropriate rest is not given, and finally becomes compassion fatigue.

That is, compassion fatigue can be defined as the emotional and behavioral responses that occur after long-term care of patients in pain. These emotions include burnout and secondary traumatic stress [24]. Burnout is closely related to the overwhelming feeling caused by a heavy administrative workload and an excessive number of patients [25], whereas secondary traumatic stress is the stress (a form of post-traumatic stress disorder) experienced by caregivers as they empathize with their patients [20]. Secondary traumatic stress affects nurses’ interpersonal interactions and communication with patients and hinders the nurses’ professional judgment, leading to medical errors [26].

According to conservation of resources theory, which was proposed by Hobfoll [27,28], individuals are motivated to conserve, protect, and accumulate the resources that they deem valuable. In particular, when individuals have fewer resources, they are more sensitive to resource reduction and adopt a more defensive mode, which is usually aggressive or even irrational, to protect themselves [29]. Compassion fatigue is the result of long-term resource depletion, leading to nurses detaching themselves from others to conserve their resources [30]. Thus, when facing patients, nurses may be inclined to use surface acting to demonstrate attitudes that conform to hospital norms. They may reduce their deep acting or expression of genuine emotions because these require proactive empathy from the nurses toward their patients [16]. Nurses experiencing compassion fatigue not only lack resources but also find themselves in a state of negative emotions [7,31]. Engaging in deep acting would require them to expend additional resources to adjust their internal cognitive feelings [32], whereas surface acting would merely involve their routine mechanical display of emotions that conform to their organizations’ norms and consume less resources [33]. Previous studies have shown that nurses with emotional exhaustion display higher levels of surface acting [34] and that employees with higher negative emotions exhibit more surface acting and less deep acting [32].

According to self-determination theory [35], humans intuitively pursue psychological growth and development. Therefore, individuals actively seek to fulfill three types of psychological needs, namely autonomy, relatedness, and competence. When these three needs are satisfied, employees have a higher level of autonomous motivation to engage in their work. By contrast, when these needs are not satisfied, they have a higher level of controlled motivation, where their behavior is driven by external norms or pressures [36]. Nurses with compassion fatigue may feel incapable of saving patients, a lack of autonomy in decision-making, and a sense of alienation in their relationships with others [37]. Such a state may prevent them from satisfying their basic needs for autonomy, relatedness, and competence, thereby reducing their intrinsic motivation. When being required to conform to the appropriate emotions required by their hospitals, the nurses may tend to engage in surface acting rather than deep acting. Also, employees’ professional identity influences their autonomous motivation [38]. Nurses with high compassion fatigue tend to take on the emotional baggage of patients with trauma, and these emotions adversely affect their professional identity and personal life [39,40] and reduce their work passion and the sympathy in their attitude toward patients [20]. A lack of professional identity and enthusiasm causes nurses to experience difficulty voluntarily demonstrating the emotions expected in their hospital [14], and they are more likely to perform surface acting and express nongenuine emotions to meet the expectations of others [41].

In accordance with this literature review, the present study posited that nurses with high compassion fatigue tend to perform surface acting rather than deep acting or expression of genuine emotions during their work. The following hypotheses were proposed:

Hypothesis 1.1: Compassion fatigue is positively associated with surface acting.
Hypothesis 1.2: Compassion fatigue is negatively associated with deep acting.
Hypothesis 1.3: Compassion fatigue is negatively associated with expression of genuine emotions.

### Moderating effect of self-compassion

When experiencing the same conditions that cause compassion fatigue, not all nurses feel pain or lose the ability to be sympathetic toward others [23]. The extent of the negative effect exerted by compassion fatigue varies with personal characteristics. Scholars have indicated that highly self-compassionate individuals have superior life satisfaction, social relationships [42], and well-being [43] and experience less pressure [44] compared with low self-compassion, who tend more to self-criticize, feel depressed or anxious, ruminative responses, suppress thought, and practice neurotic perfectionism. However, further insights are required into whether nurses with high self-compassion can avoid compassion fatigue while caring for patients.

According to Neff [42], self-compassion includes willingness to face painful experiences, being sympathetic and kind toward oneself, and an ability to understand personal mistakes and shortcomings without criticizing them, perceiving them as normal in life. Neff explored self-compassion in three dimensions. The first dimension is self-kindness, which entails being tolerant of behavior, feelings, and opinions without criticizing them. The second dimension is common humanity, which is to view unsatisfactory or negative experiences as the norm of life. No humans is perfect, and failures and mistakes are thus inevitable. The third dimension is mindfulness, which entails facing pain and negative thoughts instead of escaping from them or overly wallowing in them.

Nurses with high self-compassion may have negative emotions such as regret and pain when they face sudden changes in a patient’s condition or their death. However, through self-kindness, they can understand that one’s capabilities are limited and can thus tolerate their inability instead of undertake severe self-criticism. Such nurses may view their inability or struggles as normal, thereby remaining calm and positive when faced with difficult circumstances. They may also avoid excessively wallow in pain or negative thoughts; hence, they can provide the most effective treatment. Moreover, self-compassion enables them to be willing to learn from their mistakes, face comparable situations, and try again [45]. These qualities can help them avoid the dilemma of compassion fatigue, enabling them to provide appropriate compassion care with different patients. Studies have indicated that highly self-compassionate individuals experience less compassion fatigue [46,47].

Self-compassion helps improve an individual’s ability to respond positively [48]. It helps individuals respond favorably to work-related problems, including maintaining a balanced and neutral view of negative events as well as a compassionate attitude toward oneself, all of which help delay or interrupt the development of compassion fatigue [49]. Moreover, self-compassion is a prerequisite for extending compassion to others [50]. Gustin and Wagner [51] noted that cultivating a compassionate self and a tendency not to judge others can help increase compassion for others. Thus, self-compassion may be crucial in resisting compassion fatigue. Self-compassion helps individuals be optimistic, take the initiative to change their lives [52], and it enhances their emotional control skills and improves negative emotional states [42]. These elements are conducive to increasing positive psychological capital [53]. From the perspective of conservation of resources theory [27,28], self-compassion is considered an internal personal resource [12]. Nurses can use such resources to cope with their emotional demand at work [54]. This reduces the level of secondary traumatic stress experienced by nurses and consequently inhibits the development of compassion fatigue.

In summary, high self-compassion is likely to reduce the effect of compassion fatigue on emotional labor. Therefore, the following hypotheses were proposed:

Hypothesis2.1: Self-compassion moderates the positive relationship between compassion fatigue and surface acting. Compared with low self-compassion, high self-compassion reduces the strength of the positive correlation between compassion fatigue and surface acting.
Hypothesis2.2: Self-compassion moderates the negative relationship between compassion fatigue and deep acting. Compared with low self-compassion, high self-compassion reduces the strength of the negative correlation between compassion fatigue and deep acting.
Hypothesis2.3: Self-compassion moderates the negative relationship between compassion fatigue and expression of genuine emotions. Compared with low self-compassion, high self-compassion reduces the strength of the negative correlation between compassion fatigue and expression of genuine emotions.

**Fig 1:**
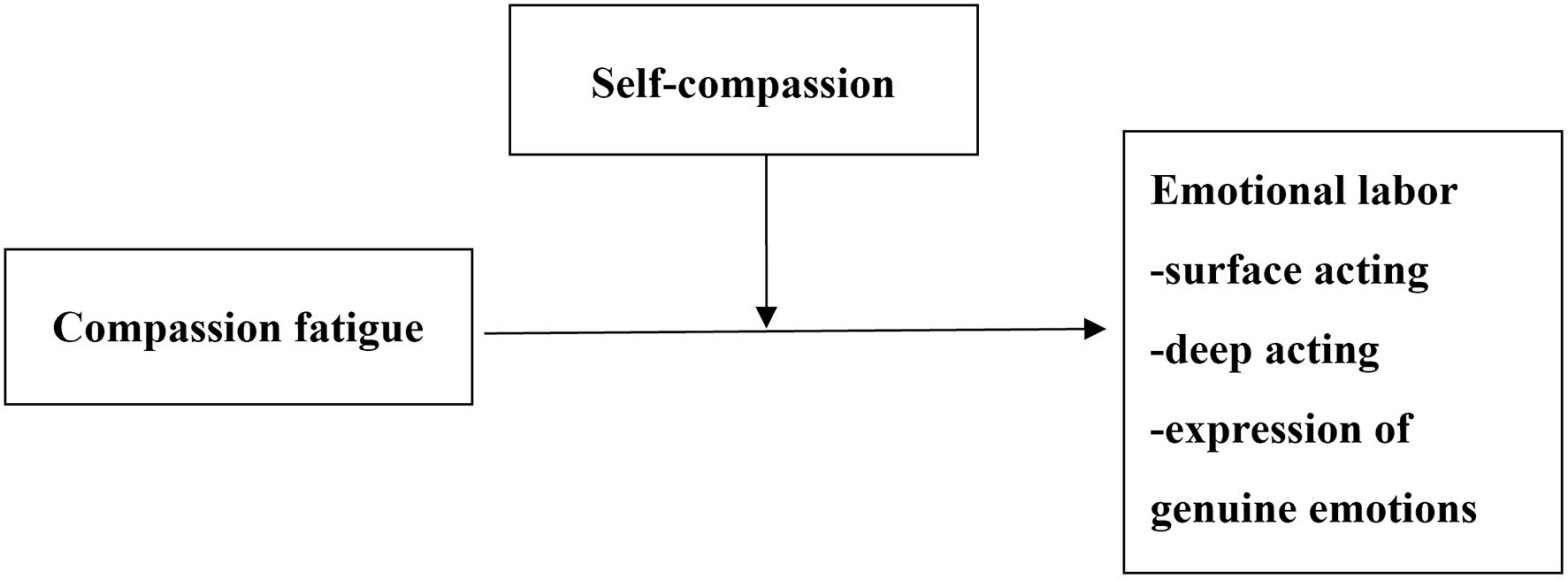
Research framework.

The research framework is presented in Fig 1.

## Materials and methods

### Study design and participants

To reduce common method bias, we adopted suggestions by Podsakoff et al. [55] and used a two-step questionnaire survey to explore whether the self-compassion effectively moderates the relationship of compassion fatigue with emotional labor. During the first stage, the participants self-evaluated their compassion fatigue, self-compassion, and demographic variables. After 2 months, the participants were re-invited to complete the second stage of the self-evaluation questionnaire, which comprised items on surface acting, deep acting, and expression of genuine emotions.

The participants of this study, selected through convenience sampling, were nurses working in medical centers, regional hospitals, and district hospitals in Central Taiwan. Participants were recruited from emergency departments, intensive care units, ward nursing units, and outpatient departments. Data collection commenced in January 2019 and finished in March 2019. Before the questionnaires were distributed, head nurses or nursing supervisors in hospitals were inquired over the phone about their willingness to participate in the survey and distribute the questionnaires to their nursing staff. To relieve the participants’ pressure in answering the questionnaires, the questionnaires were anonymous. In the two-stage questionnaire method, distributors of the questionnaires provided codes or symbols that they understood on the upper right corner of Questionnaires A and B for the same participant to facilitate matching of the questionnaires. A total of 300 questionnaire copies in each of the first and second stages were distributed, and 281 pairs of responses were retrieved; 9 pairs had invalid responses, resulting in 272 pairs of valid responses (valid response rate = 91%).

### Ethical considerations

In accordance with the Ethical Considerations, the protocol for this research was approved by the Institutional Review Board at Chung Shan Medical University Hospital, Taiwan (IRB No.: CS18130). All the participants provided written informed consent prior to completing the survey. They were informed of the purpose of the study and they have the right to withdraw from the study at any time. Their survey data were treated to guarantee confidentiality.

### Reliability and validity

Before testing the hypotheses, this study confirmed the reliability and validity of the measurement items for each construct. The study used AMOS 21 software to conduct a confirmatory factor analysis (CFA) on the measurement model, which included three or more factors as research variables, to evaluate the model fit. This involved establishing the model’s convergent validity through average variance extracted (AVE), composite reliability (CR), internal consistency [56]. Hair et al. [57] suggested that the standardized factor loadings should be greater than 0.5, and the AVE should be greater than 0.5 to ensure that the observed variables have sufficient validity to reflect the latent variables. A higher AVE value indicates a higher degree of convergent validity of the items. Guilford [58] and Hair et al. [59] suggested that the Cronbach’s alpha (CA) and composite reliability (CR) values should be greater than 0.7 to ensure internal consistency. Thus, this study performed the CFA, deleted items that failed to meet the aforementioned criteria, and reperformed the CFA to adjust the model fit. The analysis results revealed that the measurement model has convergent validity and good reliability (see Table 1).

**Table 1:** Item loading, composite reliability, average variance extracted.

Construct	Measuremnt Items	Loadings	Composite Reliability	Average Variance Extracted (AVE)
Compassion fatigue	CF1	.940	0.972	0.853
	CF2	.931		
	CF3	.910		
	CF4	.934		
	CF5	.912		
	CF6	.916		
Self-compassion	SC1	.582	0.874	0.501
	SC2	.799		
	SC3	.777		
	SC4	.636		
	SC5	.737		
	SC6	.713		
	SC7	.675		
Surface acting	SA1	.786	0.941	0.695
	SA2	.817		
	SA3	.808		
	SA4	.874		
	SA5	.783		
	SA6	.882		
	SA7	.875		
Deep acting	DA1	.770	0.902	0.700
	DA2	.888		
	DA3	.936		
	DA4	.730		
Expression of genuine emotions	EEE1	.943	0.953	0.871
	EEE2	.924		
	EEE3	.939		
Negative emotions	NE1	.519	0.889	0.506
	NE2	.610		
	NE3	.738		
	NE4	.618		
	NE5	.756		
	NE6	.770		
	NE7	.873		
	NE8	.738		

*Note*. CF: Compassion fatigue, SC: Self-compassion, SA: Surface acting, DA: Deep acting, EEE: Expression of genuine emotions, NE: Negative emotions.

### Measures

Compassion fatigue was assessed using the short version of the Compassion Fatigue Scale developed by Adams et al. [60]. This scale contained 13 items. After conducting the CFA, items with factor loadings less than 0.5 were deleted, and 6 items were retained. An example item was “Losing sleep over client’s traumatic experience.” A 5-point Likert scale was employed to score the items from 1 (*never*) to 5 (*always*). A high score indicated a high level of compassion fatigue. Evidence of criterion validity was evidenced from a sample of 263 registered nurses employed by hospitals in Taiwan [61].

Emotional labor was assessed using the Emotional Labor Scale developed by Diefendorff et al. [62]. The language in the scale was modified to suit the goal of the present study. This scale contained three dimensions, namely surface acting, deep acting, and expression of genuine emotions, and totals 14 items. The surface acting dimension had seven items. An example item was “I wear a mask to hide my actual feelings.” The deep acting dimension had four items. An example item was “I make an effort to express the emotions that I want to indicate to the patient.” The expression of genuine emotions dimension had three items. An example item was “The emotions I express are genuine when I interact with patients.” A 6-point Likert scale was used to score the items from 1 (*strongly disagree*) to 6 (*strongly agree*). A higher total score indicated that a dimension required more emotional labor. Evidence of criterion validity was collected from a sample of 315 public service employees in Taiwan [63].

Self-compassion was assessed using the Self-Compassion Scale developed by Neff [42], which contains 26 items. After conducting the CFA, items with factor loadings less than 0.5 were deleted, and 7 items were retained. An example item was “I try to see my failings as part of the human condition.” A 6-point Likert scale was employed to score the items from 1 (*strongly disagree*) to 6 (*strongly agree*). A high score indicated a high level of self-compassion. Evidence of criterion validity was collected from a sample of 772 students in Taiwan [64].

Studies have verified that organizational tenure [65] and negative emotions [32] significantly affect emotional labor. Therefore, these two variables were controlled for in this study when assessing the effect of compassion fatigue on emotional labor. Negative emotions were measured using the Positive and Negative Affect Schedule (PANAS) developed by Watson et al. [66]. The PANAS consists of 20 items; 10 items are used to measure positive emotions (e.g., joy, excitement, and enthusiasm), and the other 10 items are used to survey negative emotions (e.g., pain, guilt, and neuroticism). This study only adopted the items for evaluating negative emotions. After conducting the CFA, items with factor loadings less than 0.5 were deleted, and 8 items were retained. Participants were asked to rate the frequency of negative emotions in the past week at work on a 4-point Likert scale with endpoints at 1 (*never*) and 4 (*always*). Higher scores indicate a higher level of negative emotions. Evidence of criterion validity was collected from a sample of 555 first postgraduate year residents in Taiwan [67].

### Statistical analysis

Descriptive statistics, correlation analyses, and hierarchical regression were performed using SPSS 25.0. The demographic characteristics of nurses, compassion fatigue, self-compassion, surface acting, deep acting, and expression of genuine emotions were described using frequency, percentage, mean, and standard deviation (*SD*), which were used to understand the data distribution characteristics of each dimension. A correlation analysis was conducted to understand the relationships between the variables, and a hierarchical regression analysis was performed to test the main hypotheses. Compassion fatigue was set as the independent variable, whereas surface acting surface, deep acting, and expression of genuine emotions were set as the dependent variables. The first step of the hierarchical regression analysis involved incorporating the dependent variables and control variables (e.g., tenure and negative emotions) into the model. The second step involved adding compassion fatigue to the model, the third step involved incorporating moderator variable “self-compassion” into the model, and the fourth step involved incorporating the interaction term between compassion fatigue and self-compassion into the model. Significance of the interaction suggested that self-compassion significantly moderated the relationship between compassion fatigue and emotional labor. To clarify the nature of the interaction, groups were formed by adding or subtracting one standard deviation from the mean score of self-compassion, and an interaction graph was plotted. The statistical significance level was set at *p* < 0.05.

## Results

All participants were female nursing staff, most of whom were unmarried (173 participants; 64%), had a college education level (183 participants; 67%), held nonmanagement positions (256 participants; 94%), and worked in medical centers (174 participants; 64%). Finally, the average age and tenure of the participants was 33.06 years (SD = 9.03) and 8.55 years (SD = 7.75), respectively.

Correlation analysis revealed that compassion fatigue, self-compassion, surface acting, deep acting, and expression of genuine emotions were significantly correlated (Table 2). Compassion fatigue was positively correlated with surface acting (*r* = 0.14, *p <* 0.05), and it was negatively correlated with self-compassion (*r* = −0.21, *p <* 0.01), deep acting (*r* = −0.20, *p <* 0.01) and expression of genuine emotions (*r* = −0.35, *p <* 0.01). Self-compassion was negatively correlated with surface acting (*r* = −0.22, *p <* 0.01), and it was positively correlated with deep acting (*r* = 0.30, *p <* 0.01) and expression of genuine emotions (*r* = 0.40, *p <* 0.01).

**Table 2:** Descriptive Statistics and intercorrelations among study variables.

Variable	1	2	3	4	5
1.Compassion fatigue	(.97)				
2. Self-compassion	-.21**	(.87)			
3. Surface acting	.14*	-.22**	(.92)		
4. Deep acting	-.20**	.30**	.11	(.90)	
5.Expression of genuine emotions	-.35**	.40**	-.15*	.41**	(.95)
Mean	2.57	4.23	3.33	4.43	4.45
SD	1.25	0.63	1.07	0.78	0.99

*Note*. Cronbach’s alphas appear on the diagonal.

* *p* < .05;

** *p* < .01.

Models 1.1 and 3.1 (Table 3) illustrate that control variables accounted for a significant portion of the variance in surface acting (3%) and expression of genuine emotions (7%). Organizational tenure was positively associated with expression of genuine emotions at *β* = 0.12 and *p* < 0.05, whereas negative emotions was positively associated with surface acting at *β* = 0.15 and *p* < 0.05, and negatively associated with expression of genuine emotions at *β* = −0.23 and *p* < 0.01.

**Table 3:** Results of regression analyses on the emotional labor.

dependent variables	Surface acting				Deep acting				Expression of genuine emotions			
independent variables	step 1	step 2	step 3	step 4	step 1	step 2	step 3	step 4	step 1	step 2	step 3	step 4
	Beta	Beta	Beta	Beta	Beta	Beta	Beta	Beta	Beta	Beta	Beta	Beta
Organizational tenure	.07	.07	.07	.07	.08	.07	.08	.07	.12*	.10	.12*	.11*
Negative emotions	.15*	.13*	.11	.11	-.07	-.04	-.01	-.01	-.23**	-.17**	-.14**	-.14**
Z Compassion fatigue		.12*	.09	.11		-.18**	-.13*	-.15*		-.31**	-.24**	-.27**
Z Self-compassion			-.19**	-.19**			.27**	.27**			.34**	.33**
Z Compassion fatigue × Z Self-compassion				-.12*				.08				.15**
R^2^	.03	.04	.07	.09	.01	.04	.11	.12	.07	.16	.27	.29
Adjusted R^2^	.02	.03	.06	.07	.00	.03	.10	.10	.06	.15	.26	.28
ΔR^2^	.03*	.01*	.03**	.02*	.01	.03**	.07**	.01	.07**	.09**	.11**	.02**
F	3.47	3.67	5.33	5.19	1.59	4.12	8.48	7.14	10.28	17.04	24.42	21.59

*Note*. N = 272,

*P<0.05,

**P<0.01.

Compassion fatigue was added to the regression model for the second step. The results showed that compassion fatigue accounted for an additional 1% of the variance in surface acting at the statistical significance level of *p* < 0.05, an additional 3% of the variance in deep acting at *p* < 0.01, and an additional 9% of the variance in expression of genuine emotions at *p* < 0.01. Compassion fatigue significantly positively predicted surface acting (*β* = 0.12, *p* < 0.05) and significantly negatively predicted deep acting (*β* = −0.18, *p* < 0.01) and expression of genuine emotions (*β* = −0.31, *p <* 0.01). This means that staff with higher rates of compassion fatigue had more surface acting and less deep acting and expression of genuine emotions than did those with lower rates. These findings provide support for Hypothesis1.1, Hypothesis1.2 and Hypothesis1.3.

For the third step, self-compassion was added to the regression model. The results showed that self-compassion accounted for an additional 3% of the variance in surface acting at *p* < 0.01, an additional 7% of the variance in deep acting at *p* < 0.01, and an additional 11% of the variance in expression of genuine emotions at *p* < 0.01. Self-compassion significantly negatively predicted surface acting (*β* = −0.19, *p* < 0.01) and significantly positively predicted deep acting (*β* = 0.27, *p* < 0.01) and expression of genuine emotions (*β* = 0.34, *p* < 0.01). This means that nurses with higher self-compassion had less surface acting and more deep acting and expression of genuine emotions than did those with lower self-compassion.

For the fourth step, the multiplicative term of compassion fatigue and self-compassion was added to the regression model. The results showed that the moderated interaction term accounted for an additional 2% of the variance in surface acting at *p* < 0.05 and 2% of the variance in expression of genuine emotions at *p* < 0.01, where the interaction of compassion fatigue and self-compassion negatively predicted surface acting (*β* = −0.12, *p* < 0.05) and positively predicted expression of genuine emotions (*β* = 0.15, *p* < 0.01). We assessed the nature of this significant interaction by plotting values representing plus and minus 1 standard deviation from the means for self-compassion.

**Fig 2:**
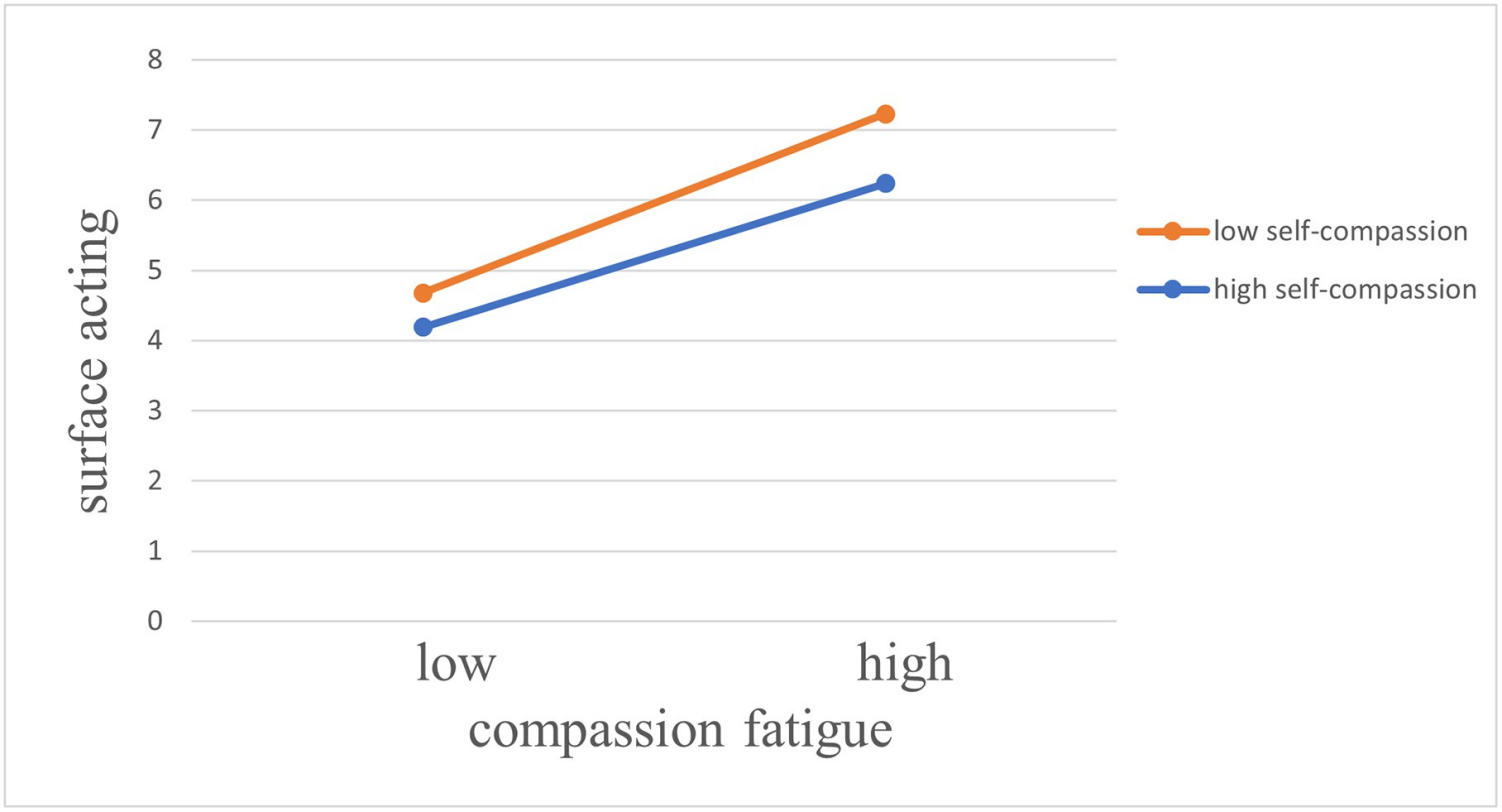
Plot of the interaction between compassion fatigue and self-compassion on surface acting.

The differential effect of high and low self-compassion on the relationship between compassion fatigue and surface acting is shown in Fig 2. The impact of compassion fatigue on surface acting was weaker (*β* = 0.32, *p* < 0.05) for employees who reported a higher self-compassion than for those who reported a lower self-compassion (*β* = 0.53, *p* < 0.01). Therefore, self-compassion effectively moderates the relationship between compassion fatigue and surface acting. Hypothesis 2.1 was supported.

**Fig 3:**
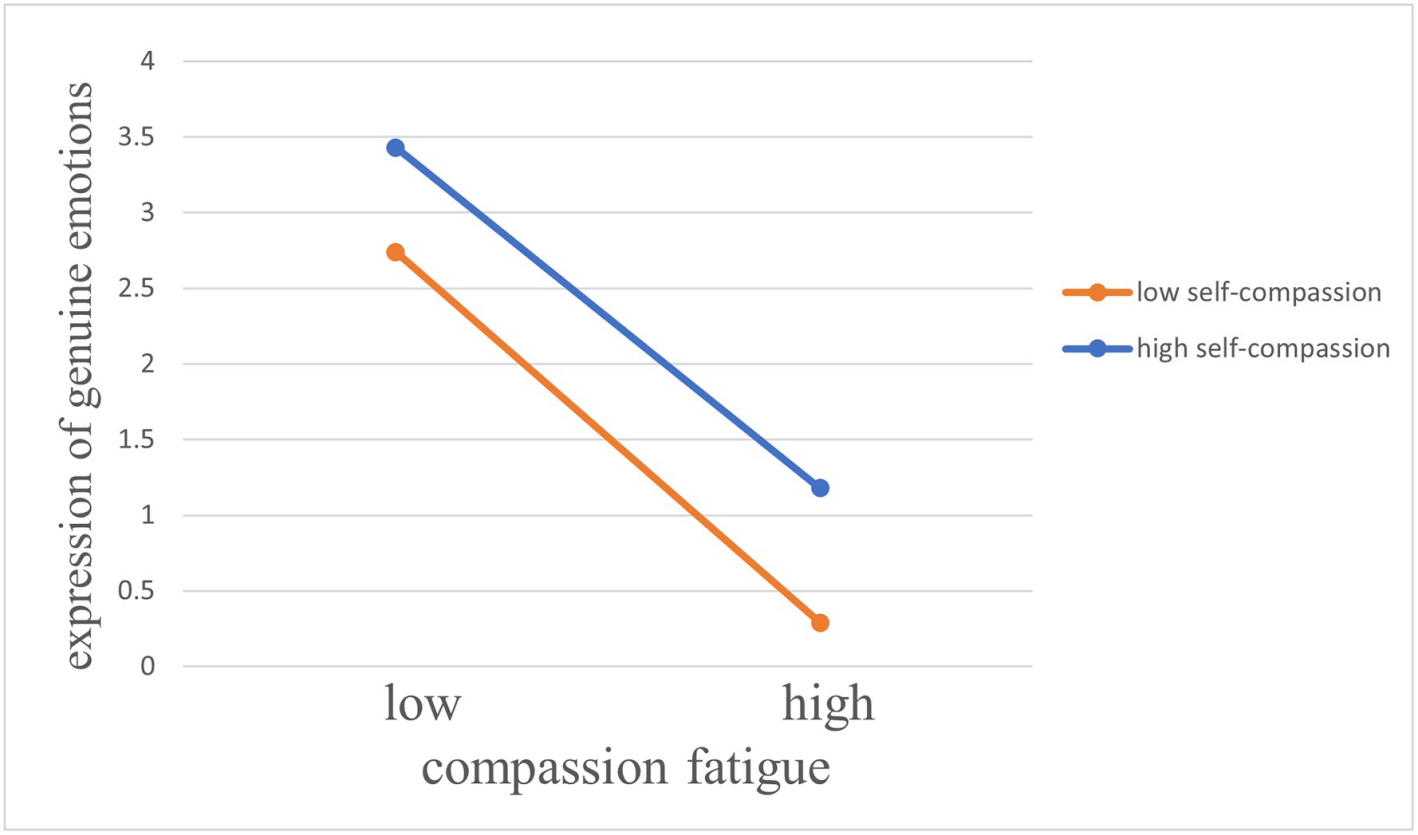
Plot of the interaction between compassion fatigue and self-compassion on expression of genuine emotions.

The differential effect of high and low self-compassion on the relationship between compassion fatigue and expression of genuine emotions is shown in Fig 3. The impact of compassion fatigue on was weaker (*β* = −0.42, *p* < 0.01) for employees who reported a higher self-compassion than for those who reported a lower self-compassion (*β* = −0.45, *p* < 0.01). Therefore, self-compassion effectively moderates the relationship between compassion fatigue and expression of genuine emotions. Hypothesis 2.3 was supported.

However, the results showed that the moderated interaction term accounted for an additional 1% of the variance in deep acting at *p* > 0.05, where the interaction of compassion fatigue and self-compassion negatively predicted deep acting (*β* = 0.08, *p* > 0.05) but not significantly effect. Accordingly, Hypothesis 2.2 wasn’t supported.

## Discussion

Existing studies have asserted that excessive emotional labor can lead to compassion fatigue in nurses [68], yet scant studies have explored whether nurses’ compassion fatigue affected their emotional labor. Therefore, this study investigated how compassion fatigue affected nurses’ emotional labor from the perspective of the conservation of resources theory and self-determination theory. Furthermore, this study examined the moderating role of self-compassion among the main variables.

The results revealed that individuals with high compassion fatigue tend to perform surface acting and are less likely to express deep acting and genuine emotions than those with low compassion fatigue. These results align with the aforementioned two theories. From the perspective of the conservation of resources theory, nurses with compassion fatigue lack internal resources and are in a state of negative emotions [31], necessitating more resource expenditure to adjust their personal cognition. By suppressing their internal emotions and mechanically displaying emotions that conform to their organizations’ norms, the nurses conserve resources [33]. Thus, nurses with high compassion fatigue exhibit more surface acting and less deep acting or expression of genuine emotions. From the viewpoint of the self-determination theory, professional identity and enthusiasm for work influence employees’ autonomous motivation [38]. Nurses with high levels of compassion fatigue may, as a result of reduced identification with their nursing profession [40] and diminished enthusiasm for nursing work, find it challenging to spontaneously express the emotional norms expected by their organizations. Consequently, they may be more inclined to adopt surface acting emotional labor to conceal their negative inner emotions, leading to less deep acting emotional labor or expression of genuine emotions [41]. This finding concurs with the findings of Barnett et al. [9], who indicated that compassion fatigue and surface acting are positively correlated.

In addition, this study verified that self-compassion effectively moderates the influence of compassion fatigue on emotional labor. Nurses experience less compassion fatigue when they are willing to face their shortcomings and failures, view them as a normal part of life, adopt a self-tolerant attitude instead of self-criticism, and avoid overly wallowing in pain or negative emotions. Previous studies have also confirmed that self-compassion can help prevent the onset of compassion fatigue [46,47]. From the perspective of the conservation of resources theory, self-compassion can be considered an internal personal resource [12] that can help individuals better address their emotional needs and regulate their internal emotions [54]. Accordingly, when such nurses are required to perform emotional labor, they are more likely to express genuine emotions, which can prevent vicious cycles of compassion fatigue and emotional dysregulation.

The results revealed that control variables such as negative emotions significantly positively predicted surface acting and significantly negatively predicted expression of genuine emotions. This finding concurs with that of Hsiao [32], who reported that when frontline service workers felt highly negative emotions, they mostly performed surface acting to display the emotions required by their organization; they rarely performed deep acting. In other words, when such employees experience negative emotions, they struggle with changing their inner emotions and are more likely to develop emotional dysregulation. Hospitals should pay attention to the impact of employee negative emotions. Besides reducing unfavorable working conditions, including long working hours, heavy workload, and rotating shifts [69], which prevent nursing staff from participating in family activities and performing their family roles and thus may lead to negative emotions in the staff, hospitals can consider practices such as increasing their salary and benefits and establishing a respectful working environment. These practices could enhance the well-being of nursing staff at work [70].

Furthermore, the result revealed that nurses with longer organizational tenure displayed more expression of genuine emotions than nurses with shorter tenure; this result was consistent with that of Karatepe and Aleshinloye [65], who reported that organizational tenure is significantly negatively correlated with emotional dissonance. Compared with less experienced employees, more experienced employees are more accustomed to their organization’s rules for emotional expression. Consequently, they can naturally express the necessary emotions at work and avoid emotional dysregulation. Hospitals can share the experience of senior nursing staff to help new nursing staff adapt quickly to the emotional norms expected by the hospital [71].

### Limitations of the study

Unlike previous studies that generally explored the impact of emotional labor on compassion fatigue [9,10], this study is the first to verify from the perspective of conservation of resources theory and self-determination theory that compassion fatigue has a significant effect on emotional labor, and that self-compassion moderates the relationship between compassion fatigue and emotional labor. However, several limitations existed in this study. First, the questionnaires distributed in this study were completed independently by nurses. Consequently, the nurses may have provided answers that concurred with social norms rather than reflected their actual feelings. Future research can distribute questionnaires to multiple types of respondents (e.g., supervisors, colleagues, and patients) to collect data and validate the proposed hypotheses. Second, although most of our hypotheses were supported, the regression model produced low *R*^2^ values, which indicated that the regression model could not explain much of the variability in emotional labor. However, many other factors may affect emotional labor, including role identity [72] and professional identity [34]. In addition to compassion fatigue, nurses may experience different types of occupational fatigue, including physical, cognitive, and emotional fatigue [4]. Studies have shown that occupational fatigue is a crucial factor affecting emotional labor [73]. Future studies can identify and control for such factors. Finally, this study employed a two-stage questionnaire survey to reduce common method biases. However, the data for the explanatory variable (i.e., compassion fatigue) and moderator variable (i.e., self-compassion) were collected and assessed during the same time period. This could have affected the correlations between variables. In the future, a three-stage questionnaire survey could be employed to further reduce common method biases (S1 File).

In medical institutions, nurses may be assigned to different wards according to their work units, and their risk of compassion fatigue may also vary. For example, nurses who work in intensive care units are more likely to encounter patient deaths than nurses working in other wards, which can result in a higher risk of compassion fatigue [74]. Future studies can investigate the relationship between compassion fatigue and emotional labor among nurses in different departments. Additionally, this study found that organizational tenure and negative emotions affect the emotional labor of nurses. Future research can explore how organizational tenure and negative emotions affect the relationship between nurses’ compassion fatigue and emotional labor.

### Practical implications

When caring for patients, nurses are often expected to exhibit high levels of compassion and care [75,76]. Compassion care helps nurses understand patients’ pain and needs, establish a good nurse-patient relationship and provide excellent quality of care [77]. However, providing excessive care for traumatized patients leads nurses to experience chronic exposure to the traumatic environment, leading to compassion fatigue [20]. The long-term accumulation of compassion fatigue has various negative impacts on hospitals and nurses, such as reduced quality of care and job satisfaction, increased turnover intention [78], and reduced job performance and organizational citizenship behavior [61].

This study showed that compassion fatigue is an essential factor affecting emotional labor. Without appropriate strategies to mitigate nurses’ compassion fatigue, nurses may engage in surface acting when adhering to hospital-mandated emotional expression, worsening their compassion fatigue and creating a vicious cycle. Determining how hospitals can alleviate the compassion fatigue of nurses is an imperative topic. A deep understanding of the factors inducing compassion fatigue must be gained before effective strategies can be designed. For example, nurses who work in intensive care units, hospice care wards, and oncology wards frequently encounter death of patients, complex disease treatments, and patients in relatively severe disease states; thus, nurses facing these patient factors develop compassion fatigue more easily than their counterparts in other units [79–81]. Nurses who are overly conscious of their inabilities or lack of experience and nurses who overly engage in patient care are also likely to experience compassion fatigue. Additionally, nurses who are assigned to care for too many patients with severe conditions or traumas are likely to experience compassion fatigue [82]. Approaches to alleviating compassion fatigue include job rotation so that nurses can switch their work environment, experience sharing by clinical nurse instructors or life instructors, and reducing the patient–nurse ratio and workload of nurses [61,83]. In stressful care situations, nurses should examine whether they are over-committed to a traumatized patient, adopt mindfulness, and effectively separate work from their personal life [84] to avoid excessive immersion in the situations.

In addition, studies have indicated that individuals with high self-compassion experience less compassion fatigue [46,47]. This study demonstrated that self-compassion moderates the relationship between compassion fatigue and emotional labor. Self-compassion can be considered an inner personal resource. Therefore, increasing self-compassion is an effective means of both alleviating and preventing compassion fatigue. Nursing education can include training in enhancing self-compassion [85], which consists of mindfulness training, compassion imagination exercises, compassion letter writing, and compassion behavior presentation, after which trainees can share their experiences and engage in discussion. Through such courses, nurses can learn how to treat themselves well at work, and how to manage emotions and negative thoughts when engaging in self-criticism. Head nurses can regularly measure the degree of self-compassion among nurses and improve related training programs to ensure that the entire nursing staff can adequately cope with compassion fatigue [86].

Andrews et al. [87] indicated that the self-compassion of nurses may be affected by the organizational environment and culture. The care of patients is often prioritized in hospitals, whereas nurse self-care is neglected. Therefore, supervisors can demonstrate self-compassion thinking and behavior patterns to encourage staff to consider self-compassion a healthy, beneficial behavior, thereby increasing their willingness to learn and cultivate such behavior. In brief, when an organizational culture encourages self-compassion, employees can learn from each other and establish a self-compassionate mentality [88].

## Conclusion

From theoretical perspectives, this study hypothesized that when nurses experience high compassion fatigue, they tend to perform surface acting and avoid deep acting and expression of genuine emotions. The proposed hypotheses were supported by the empirical results. Hospital executives should propose improvement strategies that can prevent the compassion fatigue on nurses. This study verified the moderating effect of self-compassion on the relationship between compassion fatigue and emotional labor. Hospitals can provide related training or adopt relevant practices to enhance the self-compassion ability of nurses. Sympathy toward patients is considered a core competency of nurses. However, nurses need also be sympathized with, and such sympathy can come from hospitals, supervisors, colleagues, and, most crucially, the nurses themselves.

## Supporting information

S1 File Raw data. (XLSX)
